# Portable Electronic Olfactometer for Non-Invasive Screening of Canine Ehrlichiosis: A Proof-of-Concept Study Using Machine Learning

**DOI:** 10.3390/vetsci13010088

**Published:** 2026-01-15

**Authors:** Silvana Valentina Durán Cotrina, Cristhian Manuel Durán Acevedo, Jeniffer Katerine Carrillo Gómez

**Affiliations:** 1 Multisensory Systems and Pattern Recognition Research Group, Faculty of Engineering and Architecture, University of Pamplona (UP), Pamplona 543050, Colombia; silvana.duran@unipamplona.edu.co; 2 Chemical Engineering Group, Faculty of Engineering and Architecture, University of Pamplona (UP), Pamplona 543050, Colombia

**Keywords:** canine ehrlichiosis, electronic olfactometer, MOX sensors, volatile organic compounds, machine learning, non-invasive screening

## Abstract

Canine ehrlichiosis, caused by *Ehrlichia canis*, represents a relevant challenge in veterinary medicine, particularly in resource-limited settings where access to laboratory-based diagnostics may be constrained. This pilot and exploratory study aimed to evaluate the feasibility of a portable electronic olfactometer as a non-invasive screening approach, based on the analysis of volatile organic compounds (VOCs) present in breath, saliva, and hair samples from dogs. Signals were acquired using an array of eight metal-oxide (MOX) gas sensors (MQ and TGS series). After preprocessing, principal component analysis (PCA) was applied for dimensionality reduction, and the resulting features were analyzed using supervised machine-learning classifiers, including AdaBoost, support vector machines (SVM), k-nearest neighbors (k-NN), and Random Forests (RF). A total of 38 dogs (19 PCR-confirmed infected cases and 19 controls) were analyzed, generating 114 samples evenly distributed across the three biological matrices. Among the evaluated models, SVM showed the most consistent performance, particularly for saliva samples, achieving an accuracy, sensitivity, and precision of 94.7% (AUC = 0.964). In contrast, breath and hair samples showed lower discriminative performance. Given the limited sample size and the exploratory nature of the study, these results should be interpreted as preliminary; nevertheless, they suggest that electronic olfactometry may represent a complementary, low-cost, non-invasive screening tool for future research on canine ehrlichiosis, rather than a standalone diagnostic method.

## Simple Summary

Tick-borne diseases represent a significant challenge for veterinary clinics, particularly in regions with limited access to laboratory diagnostics. Canine ehrlichiosis is one such disease and may lead to severe outcomes if not properly identified and managed. Conventional diagnostic approaches rely mainly on blood-based tests that require trained personnel, laboratory infrastructure, and invasive sampling procedures. In this pilot and exploratory study, we evaluated the feasibility of a portable electronic olfactometer as a non-invasive screening approach based on the analysis of volatile organic compounds (VOCs) collected from breath, saliva, and hair samples from dogs. The device incorporates an array of eight gas sensors, and the recorded signals are processed using computational data analysis and machine-learning techniques to identify chemical fingerprint patterns associated with infected and control animals. The results suggest that saliva samples provided the most consistent discrimination between groups. Although limited by sample size and exploratory in nature, this study indicates that electronic olfactometry may represent a complementary, low-cost tool to support non-invasive screening of canine ehrlichiosis in future veterinary research, particularly in low-resource settings.

## 1. Introduction

Canine ehrlichiosis is an infectious disease caused by *Ehrlichia canis*, an obligate intracellular bacterium of the family *Anaplasmataceae*, that infects white blood cells, particularly monocytes. It is transmitted through the bite of the brown dog tick (*Rhipicephalus sanguineus*). It can affect several members of the *Canidae* family, including dogs, wolves, coyotes, and foxes; however, dogs are the most commonly infected hosts. This disease has been reported in many regions worldwide. It is especially prevalent in warm, humid climates, where tick populations are more abundant, and it is often endemic, representing a significant clinical and economic burden for veterinary practice [1].

Ehrlichiosis is a significant concern due to its impact on animal health. Although *Ehrlichia canis* primarily affects dogs, sporadic human infections have been reported, raising epidemiological interest without establishing a definitive zoonotic role [2]. Three main diagnostic approaches are used to identify *Ehrlichia canis* in dogs: serological tests such as indirect immunofluorescence assay (IFA) and enzyme-linked immunosorbent assay (ELISA), and molecular methods such as polymerase chain reaction (PCR). These are the conventional and reference methods described in the literature and in clinical guidelines [3,4]. In addition, rapid blood-based immunochromatographic tests and microscopic examination of blood smears are commonly used as initial screening tools, particularly in low-resource clinical settings. Despite their availability, these approaches rely on blood sampling and may be limited by invasiveness, animal stress, and logistical constraints in field settings or when repeated assessments are required. Therefore, the motivation of this work is not to replace established diagnostic methods, but rather to explore a complementary, noninvasive screening approach that may assist in the early prioritization of cases and support clinical decision-making, particularly in settings with limited access to laboratory-based testing.

Globally, PCR-based studies estimate a prevalence of around 7%. In Bangladesh, a survey of 246 dogs found a PCR-positivity rate of 6.9%, and among clinically monitored confirmed cases, a case-fatality rate of 47.1% was reported [5]. In Colombia, prevalence varies by city and diagnostic method: ELISA tests in 2011 reported 83% and 80% positivity in Barranquilla and Cartagena, respectively [6]; in Medellín, a serological study in clinically suspected dogs (2012–2014) found 24.8% [7]; and in shelter dogs from the Aburrá Valley, PCR detected 2.2% positivity [8]. Regarding clinical outcomes, a hospital-based study in India (PCR confirmation and clinical follow-up) reported an overall mortality of 1.68% among all dogs evaluated and a case-fatality rate of 5.35% among positive cases, indicating that disease severity is also influenced by access to diagnostic testing and treatment [9].

Transmission of *Ehrlichia canis* occurs mainly when the brown dog tick remains attached to the host for several hours while feeding, allowing the pathogen to enter the animal’s bloodstream [10].

Canine ehrlichiosis can occur in dogs of any age or sex and may affect multiple organs with varying severity depending on the clinical stage. The most common signs include fever, lethargy, weight loss, bleeding disorders, eye problems, and neurological abnormalities. The pathogen primarily replicates within monocytes and macrophages, thereby disrupting the host immune response. Throughout the course of infection, *Ehrlichia canis* progresses through three phases: acute, subclinical, and chronic, the latter of which may lead to severe pancytopenia and be fatal if left untreated [1,11].

In this context, emerging technologies, such as electronic olfactometers, have been proposed as tools for analyzing VOCs emitted by living organisms during metabolic processes. During infection, immunological and metabolic alterations, such as inflammation, oxidative stress, and changes in cellular activity, are triggered, thereby modifying the profile of volatile compounds. As a result, electronic olfactometers can detect indirect chemical “fingerprints” associated with physiological or pathological states. These VOC patterns may serve as non-invasive biomarkers for disease screening, without aiming to replace conventional diagnostic methods, but rather to support them [12].

To our knowledge, no studies have applied electronic olfactometry specifically to canine ehrlichiosis. However, several veterinary applications support this approach. For example, VOCs collected from hair have been used to detect canine visceral leishmaniasis with a sensitivity of nearly 97% and a specificity of about 94% [13]. Electronic olfactometry has also been explored in other species, including the detection of ketosis in dairy cows, infectious bronchopneumonia in calves, and bovine tuberculosis in cattle and badgers [14,15,16]. These advances, driven by machine-learning algorithms, highlight the potential of such technologies as complementary screening tools in veterinary medicine.

Electronic olfactometers are intelligent artificial devices designed to mimic the human sense of smell. Their operation relies on the analysis of gaseous samples, with a particular focus on VOCs [17]. They consist of an array of sensors of different types, each sensitive to specific characteristics of odorant molecules, along with pattern recognition and machine-learning algorithms that interpret the generated signals [18]. These devices are generally portable, provide real-time responses, and offer good accuracy and flexibility, making them well suited for clinical or field applications [17]. Widely used sensor technologies include carbon nanofibers (CNF), conducting polymers (CP), metal oxide semiconductors (MOX), and surface acoustic wave (SAW) devices, each exhibiting distinct sensitivity characteristics and response dynamics [19].

Therefore, the present study aims to explore, from a pilot and proof-of-concept perspective, the potential of VOCs in canine ehrlichiosis. To this end, we evaluate the use of a MOX-based electronic olfactometer to differentiate between dogs infected with *Ehrlichia canis* and healthy dogs by analyzing VOCs in breath, saliva, and hair samples, using machine-learning algorithms for classification.

## 2. Materials and Methods

### 2.1. Controlled Environmental and Pre-Analytical Conditions

Sample collection was performed under controlled clinical conditions to minimize external variability. Measurements were carried out at an ambient temperature of approximately 20 °C in an indoor veterinary setting. No active control of relative humidity was implemented.

Before sample collection, dog owners were instructed to follow specific pre-analytical protocols. Animals were fasted before breath and saliva sampling to minimize the effects of recent food intake and external contaminants on VOC profiles. For hair samples, dogs had not been bathed for at least 8 days before collection to avoid interference from shampoos or other cosmetic products. During all procedures, standard biosafety measures were applied, including the use of disposable gloves, to prevent cross-contamination and ensure sample integrity. These protocols were implemented to improve the reliability and reproducibility of the measurements across infected and control groups.

**Figure 1:**
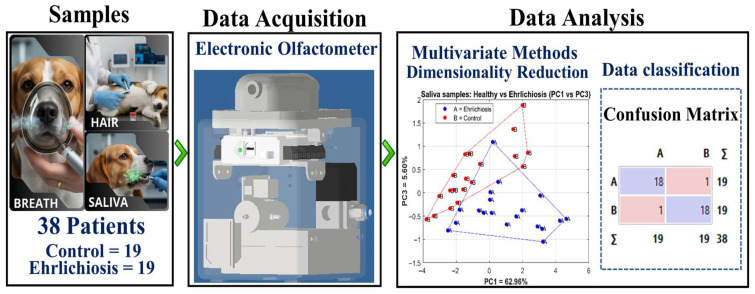
Sample acquisition and analysis workflow for dogs with Ehrlichiosis and controls.

Figure 1 presents an overview of the experimental and analytical workflow used in this study. The left panel illustrates the biological matrices collected from each dog (breath, saliva, and hair), including the total number of subjects (38 dogs: 19 *Ehrlichia canis*–positive and 19 controls). The central panel shows the electronic olfactometer used for data acquisition, in which the MOX gas sensors recorded volatile organic compounds released by each sample. The right panel summarizes the data analysis stages, including dimensionality reduction via principal component analysis (PCA) and supervised classification methods. The PCA plot illustrates the projection of saliva samples onto the principal component space (PC1 vs. PC3) for visualization purposes. The confusion matrix summarizes the classification results from a stratified 5-fold cross-validation using the support vector machine (SVM) classifier and provides an overall assessment of model performance across all folds.

In this pilot study, 38 dogs were selected: 19 with a clinical diagnosis of canine ehrlichiosis and 19 healthy controls. Dogs in the infected group were confirmed as *Ehrlichia canis*–positive by PCR testing of blood samples. Control dogs were classified as disease-free based on clinical evaluation performed by the attending veterinarian at the time of sampling.

Before sampling, the dog owners signed an informed consent form detailing the procedures, potential risks, and the study’s academic and scientific purposes. The handling of personal data was carried out in accordance with the provisions of Law 1581 of 2012 on the protection of personal information in Colombia [20].

The inclusion criteria comprised dogs of any breed, sex, or age that were either confirmed as *Ehrlichia canis*–positive by PCR testing or classified as clinically healthy based on a comprehensive veterinary examination. Exclusion criteria included aggressive behavior that precluded safe sample collection, severe clinical instability, or the absence of informed owner consent. Dogs in the infected group could be at different stages of the disease (e.g., acute, subclinical, or chronic), which may influence metabolic activity and VOC profiles. This clinical heterogeneity was intentionally accepted due to the exploratory, proof-of-concept nature of the study and is explicitly acknowledged as a limitation in the Section 4. Table 1 presents the individual characteristics of the dogs included in the list: animal code, breed, sex, and the accessibility of saliva, breath, and hair samples. Additional clinical observations are reported, together with the classification of each dog as *Ehrlichia-canis*-positive or control. This table provides an overview of the study cohort and the biological matrices analyzed for each subject. It should be clarified that, in the present study, information regarding sex(Male(M) and Female(F)), age, and weight was not available, as these data were not authorized for release by the veterinary center for ethical and administrative reasons related to data protection and owner confidentiality. The symbol “x” is used to indicate that the corresponding information or biological sample was obtained for that subject. Consequently, no statistical comparisons for these variables could be performed.

### 2.2. Design of the Electronic Olfactometer

As previously mentioned, the electronic olfactometer used in this study was designed to detect canine ehrlichiosis using samples from infected and control dogs. The system comprises eight MOX gas sensors [21], specifically from the MQ series (Winsen Electronics, Zhengzhou, China) and the TGS series (Figaro Engineering Inc., Osaka, Japan). These sensors were selected for their high sensitivity and differential response to a wide range of volatile chemical compounds. All sensors were housed in a hermetically sealed stainless-steel chamber (approximately 20 mL), a custom-built chamber for this study to ensure measurement stability and prevent cross-contamination.

**Figure 2:**
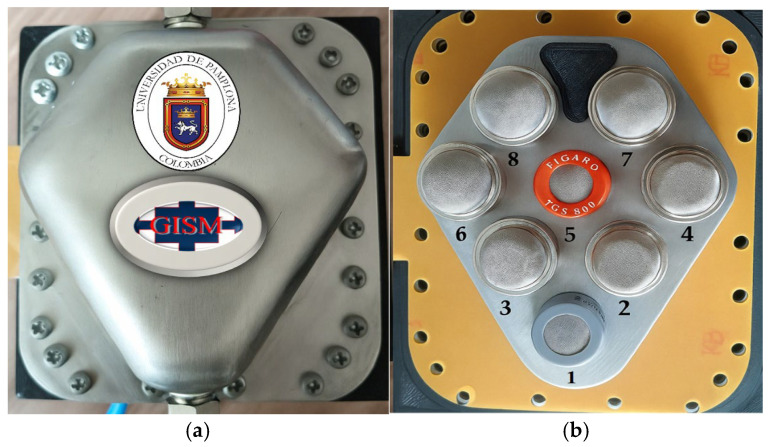
Sensor Chamber. (**a**) External view of the stainless-steel chamber; (**b**) Internal sensor configuration.

Table 2 illustrates the model specifications and the main characteristics of the sensors used, while Figure 2 shows the sealed chamber and the spatial arrangement of the units within the system.

To ensure measurement accuracy and sensor stability, a rigorous normalization and stabilization protocol was implemented. Before each sampling session, the sensors underwent a 20 min pre-heating period to reach their optimal operating temperature. For each measurement, a baseline stabilization phase was performed by flowing filtered ambient air through the chamber for 60–120 s until the sensor resistance reached a stable steady state. This procedure allows the system to compensate for sensor drift and environmental variability, ensuring that the recorded response reflects only the VOCs from the biological samples.

For sample delivery, an electropump was connected to a two-way solenoid valve. One pathway allowed the controlled delivery of the sample into the chamber, while the other was used to purge the system with clean air after each measurement. This procedure ensured proper system cleaning and prevented both sensor saturation and cross-contamination between consecutive samples.

**Figure 3:**
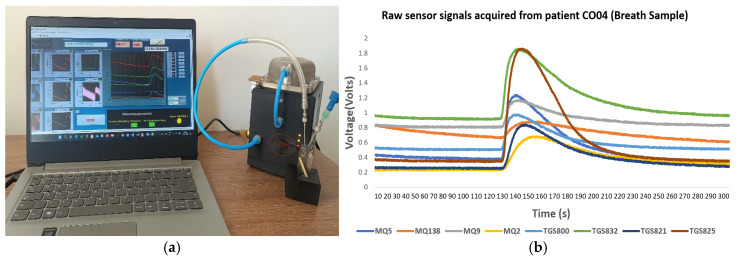
(**a**) Signal acquisition and monitoring interface of the olfactometer system, (**b**) Original real-time sensor responses recorded during breath sampling. Raw sensor signals acquired from a dog diagnosed with ehrlichiosis.

Figure 3a illustrates the signal acquisition and monitoring interface of the olfactometer system, and Figure 3b shows a raw sensor signal recorded from a canine subject with confirmed ehrlichiosis. In addition, the system enables automatic storage of collected data, facilitating subsequent processing and analysis. The patient code corresponds to a canine subject with confirmed *Ehrlichia canis* infection.

The signals from the sensors were acquired at 1 Hz, enabling continuous and stable recording of each sensor’s dynamic response during VOC exposure. These signals were acquired using an NI USB-6009 data acquisition device (National Instruments Corporation, Austin, TX, USA) [22], which enables real-time, simultaneous reading of all eight sensors in the system. The device was connected to a computer running LabVIEW 21.0.1f2 version (64-bit), which provided a graphical interface for visualizing and monitoring each sensor’s behavior throughout the measurement process.

### 2.3. Sample Collection

**Figure 4:**
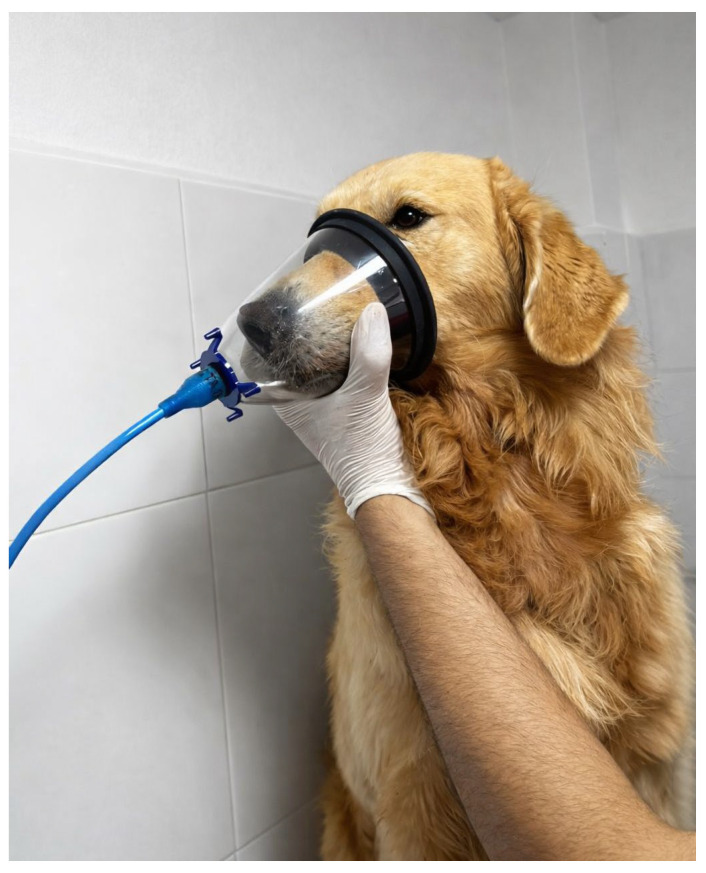
Breath sample collection process using the electronic olfactometer.

- Breath sample:

For breath sample collection, the dogs exhaled through an oxygen mask connected to the olfactometer, as shown in Figure 4, following the sampling and purging procedure described in Section 2.1.

The signals generated by the sensors were acquired and stored in the computer system and then exported in .txt format to optimize processing and reduce computational load. The experimental protocol consisted of a total cycle of 300 s (5 min) per sample, divided into four stages: system start-up (60 s), stabilization (120 s), data acquisition (20 s), and purging with clean air (100 s). This temporal scheme ensured measurement repeatability and prevented sensor saturation.

- Saliva sample

**Figure 5:**
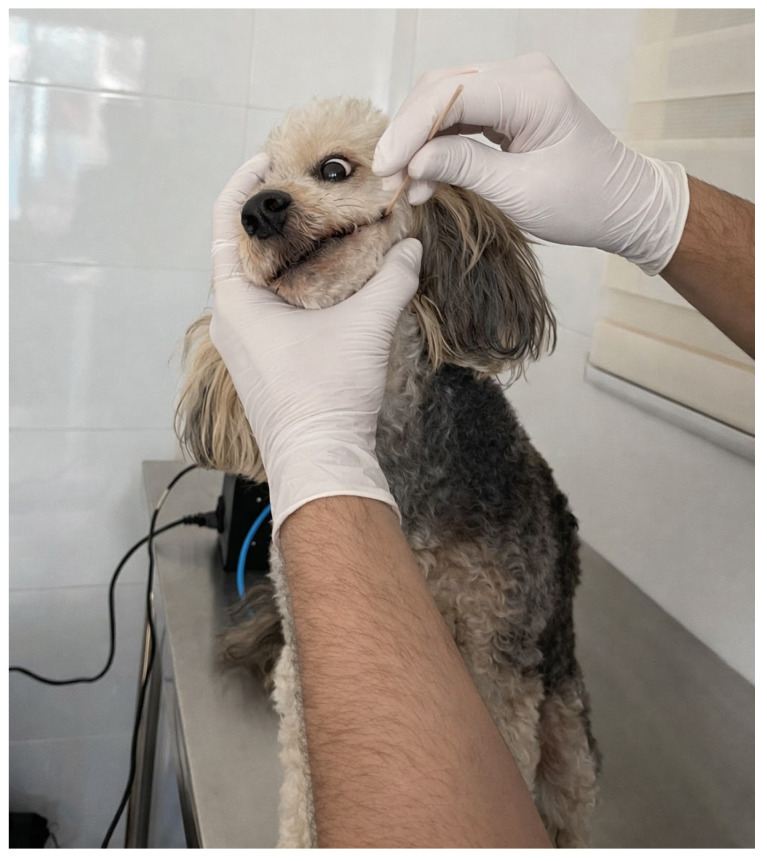
Saliva sample collection process.

Figure 5 illustrates the procedure used for saliva collection. A sterile oral swab was gently inserted into the animal’s mouth and placed between the cheek and gums for approximately 30 s to obtain a representative amount of salivary fluid.

Each swab was then placed in a sterile plastic container and stored at 4 °C until analysis.

- Hair sample

**Figure 6:**
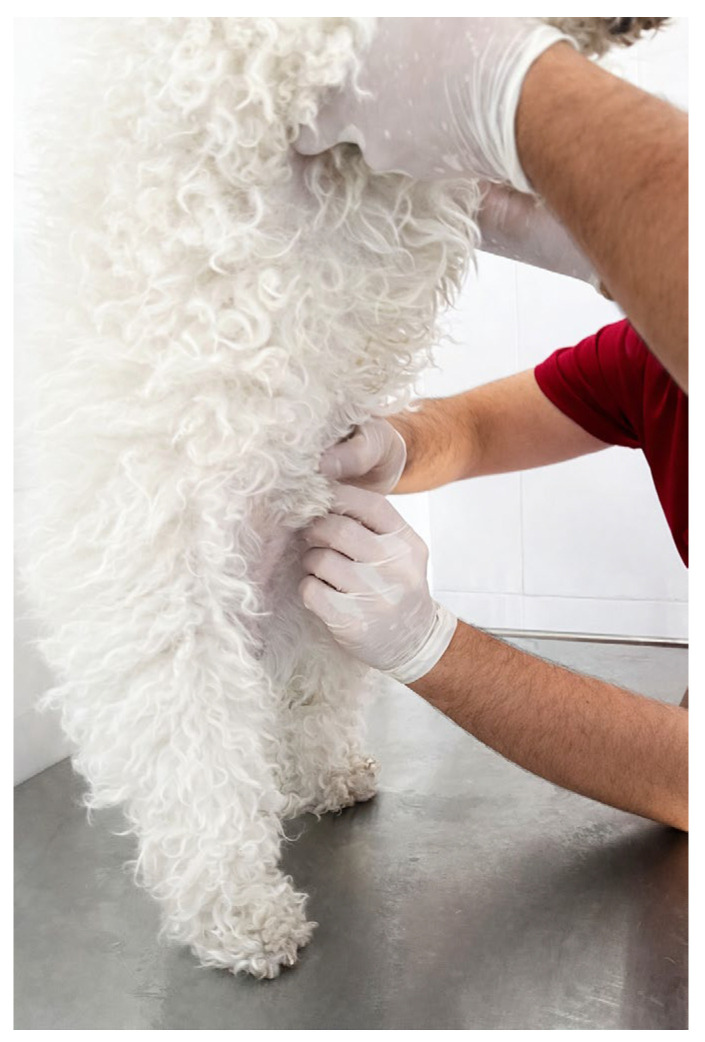
Hair sample collection process.

For hair sample collection, a small amount of hair was trimmed from the upper abdominal region (the cranial portion of the ventral abdomen). Similarly to the saliva procedure, the sample was placed in a sterile vial and stored at 4 °C until analysis. Figure 6 shows the hair sample collection procedure.

**Figure 7:**
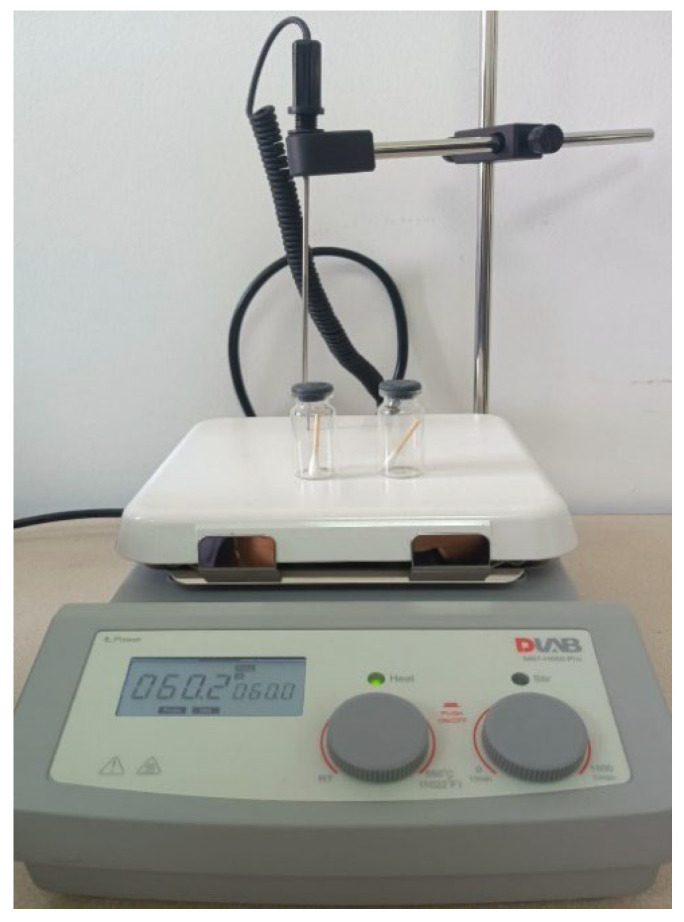
Headspace generation procedure for saliva and hair samples.

Both the hair and saliva samples were heated to 60 °C for 20 min on a DLAB MS7-H550-PRO hot plate (DLAB Scientific Co., Ltd., Beijing, China), as shown in Figure 7. This procedure generated headspace, facilitating the release of VOCs.

**Figure 8:**
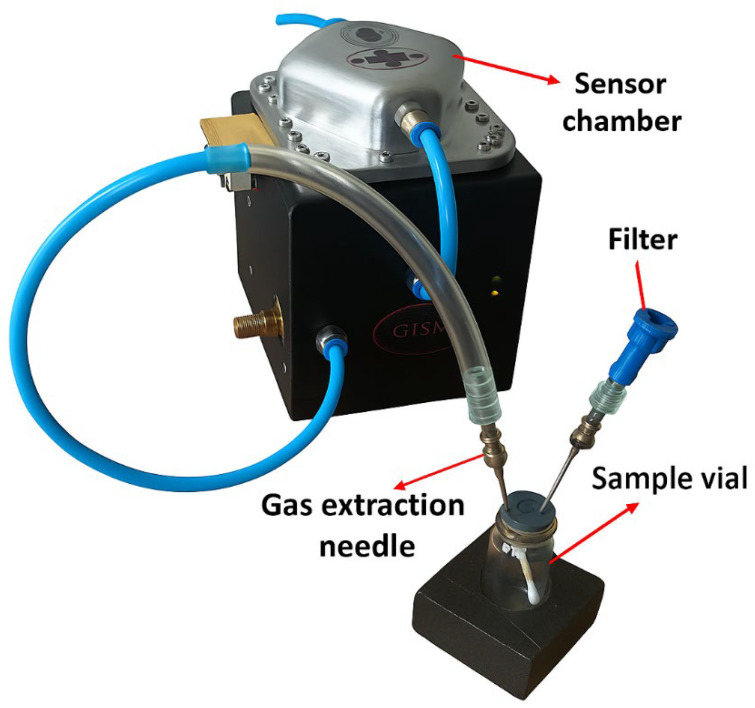
VOC extraction via direct injection into the sensor chamber.

Subsequently, the released gases were injected directly into the sensor chamber through a needle connected to the vial. To prevent contamination from ambient air, an activated carbon filter was incorporated into the vial’s septum cap, positioned next to the needle used for VOC extraction (see Figure 8). The measurements for the hair samples were carried out using the same timing intervals established for the saliva samples: 60 s for system start-up, 180 s for stabilization, 120 s for data acquisition, and 120 s for purging, resulting in a total duration of 480 s (8 min) per sample.

It is important to emphasize that this study strictly followed the biosafety protocols established for sample collection and handling in the veterinary clinic, in accordance with standard precautions aimed at preventing zoonotic transmission among veterinary personnel [23], as well as with international biosafety and biosecurity frameworks relevant to the management of biological materials [24,25]. The headspace technique was used for its simplicity and effectiveness to enable the controlled release of VOCs from canine hair samples, as previously demonstrated in diagnostic applications [13,26].

### 2.4. Data Processing Methods

This section describes the preprocessing procedures and the multivariate and machine-learning methods used to discriminate between samples from dogs with canine ehrlichiosis and those from the control group.

**Figure 9:**
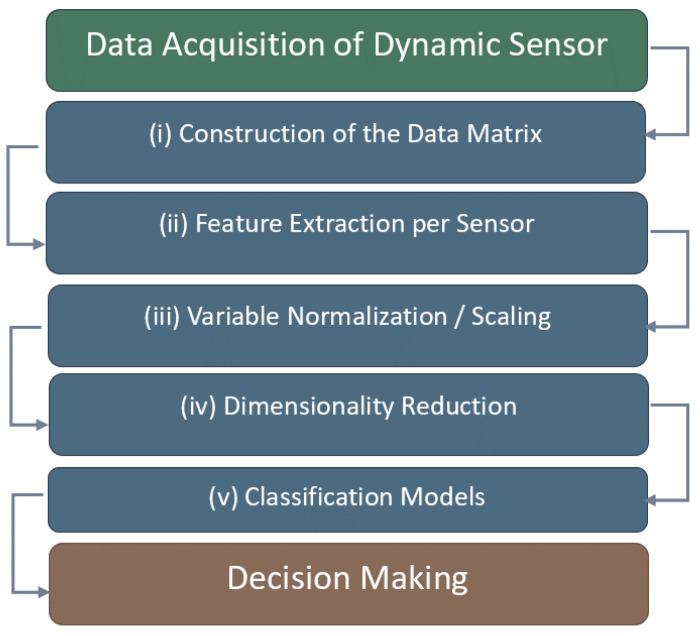
Data acquisition, processing, and classification pipeline.

The overall analysis workflow consisted of the following steps: (i) construction of the data matrix from the dynamic responses of each sensor; (ii) extraction of representative features per sensor, such as maximum values, slopes, and areas under the curve; (iii) normalization of the variables to minimize scale and intensity effects; (iv) dimensionality reduction through PCA; and (v) the use of supervised classification models (see Figure 9).

This methodological framework optimized data quality and ensured reproducibility, enabling the interpretation of relationships between sensor responses and experimental groups.

#### 2.4.1. Principal Component Analysis (PCA)

To preprocess the data, the acquired signals were first organized into a data matrix, which provided a structured representation of the data and facilitated differentiation between infected-dog and control-group samples. From the sensors’ dynamic responses, two characteristic parameters were extracted for each sensor, selected for their ability to quantitatively describe signal variations associated with VOCs linked to infection: the maximum relative change in resistance (ΔR/R_0_) and the area under the curve (AUC) during the exposure phase. The maximum change reflects the amplitude of the sensor’s response to the volatile compounds present in the sample. This parameter is calculated as the maximum variation in sensor resistance (ΔR) relative to the reference (baseline) resistance (R_0_) prior to gas exposure. In practical terms, ΔR/R_0_ quantifies the sensor’s instantaneous sensitivity and is directly related to the concentration and chemical reactivity of the detected VOCs. The following sections describe two feature extraction methods employed in this study.

1. Signal amplitude, calculated as the difference between the maximum value (*G_max_*) and the minimum value (*G_min_*) recorded during the measurement process:

(1) $$\;GA_{1}=G_{max}-G_{min}\;$$

2. Variation obtained as the difference between the final value (*G_final_*) and the initial value (*G_initial_*) of the signal:

(2) $$\;GA_{2}=G_{final}-G_{initial}\;$$

The feature matrices were then exported in .csv format and analyzed using Orange Data Mining (v3.38.0), a free and powerful Python-based platform developed at the University of Ljubljana. Orange operates through modular “widgets” that support preprocessing, model building, and evaluation, and data visualization [27].

Before analysis, each variable (sensor feature) was scaled to the interval [−1, 1] using the min–max scaling method (per variable). This transformation reduces the impact of scale differences between sensors and facilitates comparison within the component space [28].

Model performance was evaluated using 5-fold cross-validation at the sample level. To prevent information leakage, normalization and PCA analysis were applied exclusively to the training data in each fold and subsequently to the corresponding test subset.

PCA is a statistical technique that transforms a set of potentially correlated variables into a new set of uncorrelated variables known as principal components. These components capture most of the variance in the original data, enabling the identification of patterns and the visual assessment of potential clustering or overlap between classes [29,30].

#### 2.4.2. Classification Models

- k-Nearest Neighbors (k-NN)

The k-NN algorithm classifies a new sample by identifying its k-nearest neighbors in the training set and assigning it to the class with the majority vote. The nearest neighbors correspond to the k samples with the smallest distances to the sample under analysis, based on a selected metric; Euclidean distance is the most commonly used [31].

Selecting the parameter k is a critical aspect of the model: values that are too small may lead to overfitting to noise, whereas excessively large values may over smooth the decision boundary. For this reason, the optimal k is typically determined through cross-validation. k-NN is a simple, non-parametric method that does not require a complex training phase and can capture local patterns in the data. However, it is sensitive to variable scaling, making a prior normalization step necessary [32].

- Random Forest (RF)

Random Forest is an ensemble method based on decision trees. Each tree is trained on a bootstrap sample (with replacement) of the original dataset, and at each internal split, only a random subset of variables is considered. This strategy increases tree diversity and reduces the risk of overfitting. The final prediction is obtained through majority voting across all individual trees [33]. RF is characterized by robustness to noise, the ability to model nonlinear relationships, and tolerance for correlated variables. In addition, it provides estimates of variable importance and calculates out-of-bag error without a separate validation set. To achieve optimal performance, it is necessary to properly tune the number of trees, the maximum depth, and other relevant hyperparameters [34].

- AdaBoost (Adaptive Boosting)

This is a sequential and adaptive ensemble method that builds a strong classifier by combining many weak classifiers, typically very shallow decision trees or stumps. At each iteration, the algorithm increases the weights of misclassified instances and decreases those of correctly classified instances; the next classifier is then trained using these updated weights to focus on the previous errors. The final prediction is obtained by averaging the votes of all weak classifiers [35].

From a statistical perspective, AdaBoost minimizes an exponential loss function via an additive model (a sum of trees), which explains both its strong predictive performance and its sensitivity to overtraining. For this reason, it is recommended to control the number of iterations and the tree depth (or the learning rate) and to tune these parameters via cross-validation [36].

- Support Vector Machine (SVM)

This supervised classification algorithm is widely used in pattern recognition. Its goal is to separate data into classes by constructing a decision boundary that maximizes the margin to the nearest observations, known as support vectors [37,38]. The separation can be performed either in the original feature space or in a transformed space through kernel functions, allowing the model to handle nonlinear boundaries; in this study, the radial basis function (RBF) kernel was used [39].

SVM was evaluated alongside k-NN, Random Forest, and AdaBoost, and achieved the best performance across the defined metrics; therefore, it was selected for the final analysis.

- Classifier parameter settings

As mentioned, all machine-learning analyses were conducted using Orange Data Mining (version 3.38.0).

A SVM classifier was implemented as a C-SVM with a radial basis function (RBF) kernel, with C = 10 and γ (gamma) automatically determined by the software. The k-NN classifier was configured with k = 5 neighbors, employing the Euclidean distance metric and a distance-weighted voting scheme. The RF model consisted of 22 decision trees, with one attribute selected at each split, a minimum of five samples per node, and replicable training enabled. On the other hand, AdaBoost was implemented using decision trees as base estimators, with 50 estimators, a learning rate of 1.0, and a fixed random seed (seed = 2) to ensure reproducibility (see Table 3). All remaining parameters were kept at their default values, and no hyperparameter optimization was performed, in accordance with the exploratory, proof-of-concept nature of this study.

### 2.5. Confusion Matrix

The confusion matrix is a fundamental tool for evaluating the performance of classification models. This matrix compares the algorithm’s predictions with the true labels, organizing the results into four main categories: true positives (TP), false positives (FP), true negatives (TN), and false negatives (FN) [40].

Each element of the matrix enables the calculation of metrics such as precision, sensitivity (recall), F1-score, specificity, and accuracy, allowing for a more comprehensive and transparent analysis of model behavior [41].

In this study, confusion matrices were generated for each biological sample type (breath, saliva, and hair), enabling visualization of the distribution of correct and incorrect classifications for each case. These matrices were obtained by applying the evaluated classifiers to a stratified 5-fold cross-validation scheme that preserves class proportions within each fold. In each iteration, the model was trained on four folds and validated on the remaining one. Based on TP, FP, TN, and FN, the classification metrics were calculated and subsequently reported as the mean ± standard deviation across the five folds.

The metrics used in this study are described below.

- Accuracy: Proportion of correct predictions relative to the total number of samples.

(3) $$Accuracy=\frac{TP+TN}{TP+TN+FP+FN}\;$$

- Precision: Percentage of samples predicted as positive that are actually positive (controls for FP).

(4) $$\;Precision=\frac{TP}{TP+FP}\;$$

- Sensitivity (Recall): Percentage of actual positive cases correctly identified (controls for FN).

(5) $$Sensitivity=\frac{TP}{TP+FN}\;$$

- Specificity: Percentage of actual negative cases correctly identified.

(6) $$Specificity=\frac{TN}{TN+FP}\;$$

- F1-score: Harmonic mean of precision and sensitivity (balances false positives and false negatives).

(7) $$F1=\frac{2*Precisi\mathrm{on}*Sensitivity}{Precision+Sensitivity}\;$$

- ROC Curve: The ROC curve plots the true positive rate (TPR) against the false positive rate (FPR) as the decision threshold varies.

The AUROC (Area Under the ROC Curve) quantifies the model’s discriminative ability; values close to 1 indicate excellent class separation.

## 3. Results

### 3.1. Results of Multivariate Analysis: PCA

**Figure 10:**
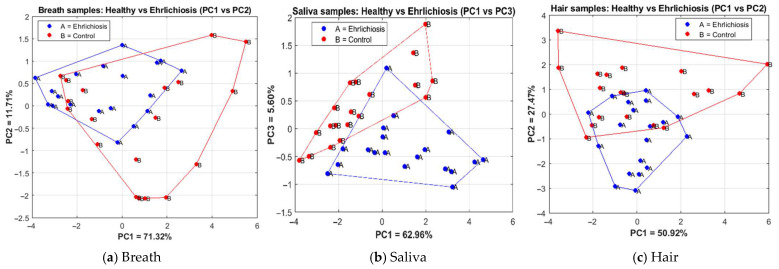
PCA plots showing the discrimination between dogs with ehrlichiosis. (**a**) Breath samples projected onto PC1 and PC2, (**b**) Saliva samples projected onto PC1 and PC3, and (**c**) Hair samples projected onto PC1 and PC2. The percentage of variance explained by each principal component is indicated on the corresponding axes.

Figure 10 shows the results of applying PCA, in which the samples are visualized in a reduced space defined by the most representative components: those with the highest explained variance and the most significant visual separation between classes. The plots show the discrimination results for breath samples (Figure 10a), saliva samples (Figure 10b), and hair samples (Figure 10c).

Each PCA includes samples from 38 dogs (19 infected and 19 control), with one sample per biological matrix per dog, yielding 38 samples per PCA plot. Because multiple biological matrices were collected from the same individuals, the PCA results should be interpreted as exploratory visualizations rather than independent statistical analyses.

- For breath samples, the explained variance was PC1 = 71.32% and PC2 = 11.71%, with a cumulative variance of PC1–PC2 = 83.03%.
- For saliva samples, PC1–PC3 was represented because it provided a clearer visual separation than PC1–PC2; the variances were PC1 = 62.96%, PC2 = 22.63%, and PC3 = 5.60%, with a cumulative variance of PC1–PC3 = 91.19%.
- For hair samples, the explained variance was PC1 = 50.92% and PC2 = 27.47%, with a cumulative variance of PC1–PC2 = 78.39%.

In all three cases, a tendency toward class clustering was observed, with partial overlap among groups. The presence of overlap indicates that complete separation between classes was not achieved within the reduced PCA space.

### 3.2. Results of the Classification Models

Table 4 summarizes the classifiers’ performance for each sample type, reporting the mean ± standard deviation (SD) of AUC, Accuracy, F1-score, Precision, Recall, and Specificity obtained through stratified 5-fold cross-validation. At this stage, the AdaBoost, SVM (RBF), k-NN, and Random Forest models were trained and evaluated following the same validation protocol, allowing a consistent comparison of their performance across folds.

It should be noted that classification performance metrics are reported at the sample level rather than at the individual-dog level, which may overestimate performance due to repeated measurements per subject.

**Figure 11:**
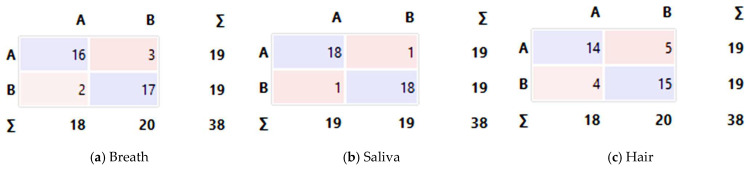
Confusion matrices obtained from the SVM classifier. (**a**) Breath samples classification (**b**), Saliva samples classification, and (**c**) Hair samples classification.

Overall, SVM achieved the highest accuracy across the three modalities, consistently outperforming the other classifiers. Figure 11 presents the confusion matrices obtained for the SVM classifier evaluated using cross-validation, with predictions aggregated across folds. A = Ehrlichia; B = Control. The matrices are read by rows (true class) and columns (predicted class).

For breath samples, SVM reached AUC = 0.884, CA = 86.8%, F1 = 86.8%, precision = 86.9%, recall = 86.8%, and specificity = 86.8%. For saliva samples, performance was higher, with AUC = 0.964, CA = 94.7%, F1 = 94.7%, Precision = 94.7%, recall = 94.7%, and specificity = 94.7%. For hair samples, SVM yielded AUC = 0.798, CA = 76.3%, F1 = 76.3%, precision = 76.4%, recall = 76.3%, and specificity = 76.3%.

- Breath (Figure 11a). The model correctly classified 16 infected dogs (TP) and 17 healthy dogs (TN). There were three false negatives (FN), in which infected dogs were predicted as healthy, and two false positives (FP), in which healthy dogs were predicted as infected.
- Saliva (Figure 11b). This modality yielded the best performance, with 18 TP and 18 TN, and only 1 FN and 1 FP. These results demonstrate a strong discriminative capability for this sample type.
- Hair (Figure 11c). The model correctly identified 14 TP and 15 TN but produced 5 FN and 4 FP, indicating lower accuracy and greater inter-class confusion.

Taken together, these results indicate that saliva samples yielded the highest discriminative performance in this exploratory study, followed by breath samples, whereas hair samples exhibited lower classification performance.

## 4. Discussion

The results of this pilot study confirm that the electronic olfactometer can distinguish between dogs with canine ehrlichiosis and healthy controls using VOC profiles obtained from breath, saliva, and hair samples. To our knowledge, this is the first exploratory study to apply electronic olfactometry to the screening for canine ehrlichiosis using non-invasive biological matrices. Overall, the findings indicate that this approach may provide useful discriminatory information, particularly for saliva samples, supporting its potential role as a complementary screening tool in resource-limited veterinary settings.

The MOX sensors used in this study were not selective for individual compounds but responded to complex mixtures of VOCs, generating a global olfactory fingerprint that depended on the biological matrix. Therefore, the system did not identify disease-specific VOCs but instead discriminated among samples using pattern recognition and machine learning.

Multivariate analysis using PCA revealed a general tendency toward class clustering across the three biological matrices, although partial overlap between infected and control groups was consistently observed. This overlap likely reflects the complex and heterogeneous nature of canine ehrlichiosis, as well as inter-individual metabolic variability. Given the lack of detailed clinical staging, quantitative PCR load, and antibiotic treatment history, the sources of overlap cannot be definitively attributed to specific biological or diagnostic factors and should therefore be interpreted with caution.

Regarding supervised classification, SVM achieved the best overall performance across all sample types, with the highest accuracy observed for saliva samples, followed by breath and hair. While similar performance trends have been reported in electronic olfactometry studies for other veterinary diseases, such comparisons should be interpreted cautiously due to differences in biological matrices, sensor configurations, experimental protocols, and cohort characteristics. In this context, the present results are intended to demonstrate feasibility rather than direct comparability with previously published diagnostic performances.

Among the evaluated biological matrices, saliva emerged as the most informative sample type in terms of both PCA visualization and classification metrics. This may be explained by the closer association of salivary metabolites with systemic physiological processes. Breath samples also provided relevant discriminatory information, although with greater variability, potentially influenced by differences in breathing patterns and sampling conditions. Hair samples showed lower discriminative performance, possibly due to dilution of disease-related VOCs by environmental or skin-derived compounds and variability in hair volume across individuals.

From a clinical perspective, the findings indicate that a compact array of eight MOX sensors, combined with machine-learning algorithms, can extract VOC-based patterns associated with canine ehrlichiosis from non-invasive samples. However, this approach is not intended to replace established diagnostic methods such as hematological analysis, serology, or PCR. Instead, it may serve as a supportive screening tool to assist clinical decision-making, particularly in exploratory studies, repeated monitoring, or veterinary practices with limited access to laboratory infrastructure.

Several limitations of this study must be acknowledged. First, the relatively small sample size limits the statistical power and the generalizability of the results. Second, although multiple biological matrices were collected from the same individuals, classification performance was evaluated at the sample level rather than at the individual-dog level, which may introduce within-subject dependence. Third, the study population comprised only dogs with confirmed ehrlichiosis and clinically healthy controls, excluding dogs with other inflammatory or infectious conditions that could also influence VOC profiles. In addition, clinical heterogeneity in disease stage and presentation was intentionally accepted to assess the electronic olfactometer’s performance under realistic veterinary conditions. While this approach limits stratified analysis, it provides insight into the system’s robustness in real-world scenarios and is appropriate for an exploratory, proof-of-concept study.

Finally, external validation, individual-level validation strategies, and control of potential confounding clinical variables were beyond the scope of this pilot study and should be addressed in future work.

## 5. Conclusions

The results of this pilot proof-of-concept study highlight the potential of the electronic olfactometer as a complementary, non-invasive screening tool for canine ehrlichiosis. Among the evaluated biological matrices, saliva analysis showed the best performance, with the SVM classifier achieving an accuracy of 94.7%, an AUC of 0.964, and high F1-score, precision, and sensitivity values. Furthermore, these results indicate strong discriminative capability in distinguishing infected dogs from control dogs, using PCR-confirmed cases as the clinical reference.

This work is the first to investigate a portable electronic olfactometer for non-invasive screening of canine ehrlichiosis. Saliva showed the highest classification performance, while breath and hair also provided consistent results (86.8% and 76.3% accuracy, respectively). These findings indicate that the system maintains reasonable discriminative capability across different biological matrices, which may be advantageous in field and low-resource settings where sample availability varies.

Overall, the findings suggest that a system based on a compact array of MOX sensors, combined with machine-learning algorithms, can provide useful information to support noninvasive screening for canine ehrlichiosis. 

Finally, it is essential to emphasize that the results should be interpreted as preliminary, given the limited sample size and the study’s pilot nature, where future work should focus on expanding the study population to include more diverse cohorts and incorporating complementary analytical techniques, such as gas chromatography–mass spectrometry (GC–MS) and electronic tongue systems. These efforts will enable a more detailed characterization of disease-associated volatile compounds and further strengthen the validation and robustness of the proposed system.

## Figures and Tables

**Table 1:** Individual characteristics of the dogs in the study.

Code	Breed	Sex	Saliva	Breath	Hair	Observations	Ehrlichiosis	Control
EC00	Golden Retriever	M	x	x	x	-		x
EC01	Poodle	M	x	x	x			x
CO02	Poodle	F	x	x	x		x	
CO03	Yorkshire Terrier	M	x	x	x		x	
CO04	Border Collie	M	x	x	x		x	
EC05	Schnauzer	F	x	x	x			x
EC06	French Bulldog	F	x	x	x			x
CO07	Mixed-breed	M	x	x	x		x	
EC08	Mixed-breed	F	x	x	x	During hysterectomy treatment, she was very restless.		x
EC09	Pinscher	F	x	x	x			x
EC10	Schnauzer	F	x	x	x			x
CO11	Siberian Husky	M	x	x	x		x	
CO12	Mixed-breed	M	x	x	x		x	
EC13	Schnauzer	F	x	x	x			x
CO14	Mixed-breed	F	x	x	x		x	
EC15	Poodle	M	x	x	x			x
CO16	Pitbull	F	x	x	x		x	
EC17	Mixed-breed	M	x	x	x			x
CO18	Mixed-breed	F	x	x	x	Gastroenteritis	x	
EC19	Poodle	F	x	x	x			x
EC20	Mixed-breed	M	x	x	x			x
CO21	Mixed-breed	F	x	x	x	Undergoing blood transfusion and splenomegaly	x	
CO22	Schnauzer	M	x	x	x	Treatment—lipoma removal	x	
EC23	Poodle	M	x	x	x			x
CO24	Mixed-breed	F	x	x	x	Chronic otitis	x	
EC25	Mixed-breed	M	x	x	x			x
EC26	Jack Russell	M	x	x	x			x
CO27	Siberian Husky	F	x	x	x		x	
EC28	Pug	M	x	x	x			x
EC29	Cocker Spaniel	F	x	x	x			x
CO30	Mixed-breed	F	x	x	x	Treatment for tick infestation—hepatitis	x	
EC31	Poodle	M	x	x	x			x
CO32	Chihuahua	F	x	x	x		x	
EC33	Poodle	F	x	x	x			x
EC34	French Bulldog	M	x	x	x			x
CO35	Shih Tzu	F	x	x	x		x	
CO36	Schnauzer	M	x	x	x		x	
CO37	Golden Retriever	M	x	x	x		x	

**Table 2:** MOX gas sensors for the Electronic Olfactometer.

No.	Sensor/Model	Target Gases/Primary Application
1	MQ5	Natural gas, LPG
2	MQ138	Toluene, Acetone, Ethanol, Formaldehyde
3	MQ9	Carbon monoxide (CO), Flammable gases
4	MQ2	Propane, Methane, Alcohol, Hydrogen
5	TGS800	Air contaminants such as Hydrogen, Ethanol, CO, Methane, Isobutane
6	TGS832	Chlorofluorocarbons
7	TGS821	Alcohol vapors, Ammonia, Hydrogen
8	TGS825	Hydrogen sulfide

**Table 3:** Model parameter configuration.

Classifier	Parameter	Value
SVM	Type	C-SVM
	Kernel	RBF
	C (Cost)	10
	γ (gamma)	Auto
k-NN	Number of neighbors (k)	5
	Distance metric	Euclidean
	Weighting scheme	Distance-weighted
Random Forest	Number of trees	22
	Attributes per split	1
	Minimum samples per node	5
	Replicable training	Enabled
AdaBoost	Base estimator	Decision Tree
	Number of estimators	50
	Learning rate	1.0
	Random seed	2

**Table 4:** Evaluation metrics (mean ± SD) for each sample type obtained using stratified 5-fold cross-validation.

Sample	Model	AUC (Mean ± SD)	Accuracy (% ± SD)	F1 (% ± SD)	Precision (% ± SD)	Recall (% ± SD)	Specificity (% ± SD)
Breath	AdaBoost	0.658 ± 0.381	65.8 ± 32.5	64.6 ± 34.2	68.3 ± 25.2	65.8 ± 26.4	65.8 ± 22.9
	SVM	0.884 ± 0.155	86.8 ± 13.7	86.8 ± 20.6	86.9 ± 15.2	86.8 ± 14.4	86.8 ± 14.1
	k-NN	0.751 ± 0.222	68.4 ± 31.9	68.4 ± 27.8	68.4 ± 25.5	68.4 ± 27.3	68.4 ± 30.8
	Random Forest	0.878 ± 0.112	76.3 ± 23.5	75.9 ± 23.7	78.3 ± 21.7	76.3 ± 25.4	76.3 ± 21.2
Saliva	AdaBoost	0.658 ± 0.394	65.8 ± 27.7	65.8 ± 23.0	65.8 ± 28.4	65.8 ± 32.5	65.8 ± 30.6
	SVM	0.964 ± 0.052	94.7 ± 11.4	94.7 ± 14.7	94.7 ± 12.3	94.7 ± 11.6	94.7 ± 10.1
	k-NN	0.803 ± 0.137	84.2 ± 15.4	83.8 ± 15.7	84.2 ± 18.5	83.8 ± 16.7	84.2 ± 20.7
	Random Forest	0.776 ± 0.260	76.3 ± 21.3	75.9 ± 22.5	78.3 ± 22.8	76.3 ± 24.7	76.3 ± 25.0
Hair	AdaBoost	0.553 ± 0.489	55.3 ± 41.3	54.5 ± 40.3	55.7 ± 38.5	55.3 ± 40.5	55.3 ± 35.0
	SVM	0.798 ± 0.226	76.3 ± 16.3	76.3 ± 20.3	76.4 ± 19.2	76.3 ± 24.2	76.3 ± 22.0
	k-NN	0.723 ± 0.288	68.4 ± 29.5	68.3 ± 29.7	68.6 ± 28.5	68.4 ± 27.3	68.4 ± 29.3
	Random Forest	0.737 ± 0.261	68.4 ± 30.5	68.3 ± 28.8	68.6 ± 29.4	68.4 ± 28.3	68.4 ± 27.3

## Data Availability

The data presented in this study are available on request from the corresponding authors. The data are not publicly available due to privacy restrictions on clinical records for privately owned animals.

## Abbreviations

The following abbreviations are used in this manuscript:

VOCs	Volatile Organic Compounds
MOX	Metal Oxide Gas Sensor
PCA	Principal Component Analysis
SVM	Support Vector Machine
k-NN	k-Nearest Neighbors
RF	Random Forest
GC–MS	Gas Chromatography–Mass Spectrometry
IFA	Indirect Immunofluorescence Assay
ELISA	Enzyme-Linked Immunosorbent Assay
PCR	Polymerase Chain Reaction
CNF	Carbon Nanofiber
CP	Conducting Polymer
SAW	Surface Acoustic Wave
DAQ	Data Acquisition
ROC	Receiver Operating Characteristic
AUC	Area Under the ROC Curve
CA	Classification Accuracy
TP	True Positive
TN	True Negative
FP	False Positive
FN	False Negative
